# Effects of Degraded Optical Conditions on Behavioural Responses to Alarm Cues in a Freshwater Fish

**DOI:** 10.1371/journal.pone.0038411

**Published:** 2012-06-20

**Authors:** Lynn Ranåker, P. Anders Nilsson, Christer Brönmark

**Affiliations:** Aquatic Ecology, Department of Biology, Lund University, Lund, Sweden; UC Santa Barbara, United States of America

## Abstract

Prey organisms often use multiple sensory cues to gain reliable information about imminent predation threat. In this study we test if a freshwater fish increases the reliance on supplementary cues when the reliability of the primary cue is reduced. Fish commonly use vision to evaluate predation threat, but may also use chemical cues from predators or injured conspecifics. Environmental changes, such as increasing turbidity or water colour, may compromise the use of vision through changes in the optical properties of water. In an experiment we tested if changes in optical conditions have any effects on how crucian carp respond to chemical predator cues. In turbidity treatments we added either clay or algae, and in a brown water colour treatment we added water with a high humic content. We found that carp reduced activity in response to predator cues, but only in the turbidity treatments (clay, algae), whereas the response in the brown water treatment was intermediate, and not significantly different from, clear and turbid water treatments. The increased reliance on chemical cues indicates that crucian carp can compensate for the reduced information content from vision in waters where optical conditions are degraded. The lower effect in brown water may be due to the reduction in light intensity, changes in the spectral composition (reduction of UV light) or to a change in chemical properties of the cue in humic waters.

## Introduction

Predation is an important structuring force in freshwater ecosystems and acts as a strong selection pressure for anti-predator adaptations in prey organisms. Prey may decrease risk of predation by having e.g. chemical or morphological defences, changing their habitat use, or by adjusting their activity pattern. A reduced activity of a prey individual generally decreases predator encounter rates [1], [2] and this should affect the spatio-temporal probability of predation. However, it is well known that a behavioural response to predation threat also results in a cost of lost opportunities to engage in other activities, e.g. foraging, territorial defence or mating [3], [4]. Thus, natural selection should favour the evolution of prey ability to accurately identify and quantify predation threat to avoid erroneous behavioural decisions.

Freshwater fish have a well-developed visual system and it has been argued that vision is their primary source of information about the environment (e.g. [5], [6]). However, fish may also use other senses to detect predator presence, including the lateral line system [7], [8] or chemoreception [9], [10], [11], [12]. Although fish may primarily rely on one source of information, it is likely that they integrate multiple cues to increase accuracy in predation risk assessment, and several studies have shown additive effects of visual and chemical cues on different threat-sensitive behaviours in prey fish [13], [14], [15], [16]. In a conceptual model, the sensory compensation model, Hartman and Abrahams [17] assumed that fish primarily use visual cues to evaluate predation threat and suggested that the threshold of alarm cue concentration necessary to elicit a behavioural response was dependent on predation risk and the quality of the visual information. They predicted that the threshold concentration should decrease in response to reduced visual information when risk of predation was low, and in an experiment with fathead minnows they were able to show that at low risk minnows displayed fright behaviours in response to chemical cues only when the information from visual cues was reduced due to increasing turbidity (created by adding clay).

The optical environment of the water should affect the importance of visual information for freshwater fish, and a number of studies have shown that, for example, increasing turbidity results in decreased reaction distance between predator and prey [18], [19], [20], [21]. The primary factors that affect the optical properties of water include backscattering of light by suspended particles, and absorption or attenuation of incident light by algae and dissolved organic matter, and these factors are in turn affected by environmental changes. Erosion, caused by e.g. altered land use or precipitation patterns, increases the concentration of inorganic particles, such as clay, that scatter light and deteriorate visibility conditions. Eutrophication, that has historically been one of the major threats to freshwater systems [22], benefits algal growth, and algae both scatter light and absorb red and blue wavelengths. Humic substances have in recent years been recognised as a growing threat to freshwater systems [23], [24], and although the exact cause behind the increase in humic substance concentrations is still debated (e.g. [24], [25]), these dissolved organic substances attenuate shorter wavelengths and shift the spectral range of available light towards red or even infrared, without scattering light. Clay, algae and humic substances hence all drastically deteriorate the visual conditions in water, but by different mechanisms with different effects on optical conditions, and may therefore have different effects on the ability of fish to use visual cues, and thereby also affect their reliance on chemical cues in a visually degraded environment.

In this study, we focus on how water colour (humic substances) and turbidity (clay and algae) affect fright responses in a freshwater fish, the crucian carp *Carassius carassius*. From earlier studies we know that crucian carp react to chemical cues from piscivorous pike (*Esox lucius*) in clear water by changing morphology and behaviour [9]. According to the sensory compensation model, an increased reliance on chemical cues with deteriorated optical properties should increase their behavioural responses. Furthermore, if different environmentally induced optical degradations (algae, clay, brown water) differ in how they affect vision in crucian carp, we predict differences in the behavioural responses to chemical cues in different media. Although the relative effects of absorbance and scattering of light on fish visual accuracy and reliability are poorly investigated in this context, we hypothesized that scattering light, which diffuses the visual image, should decrease the reliability of vision more than changes in the spectral range due to absorption of specific wavelengths. Therefore, we predicted that crucian carp should show a larger behavioural response to alarm cues in turbid water than in clear or brown water.

## Materials and Methods

### Fish

Crucian carp *Carassius carassius* (total length: 11.5±1 cm, mean±S.D.) were caught in a pond in Lund, Southern Sweden, using a trap, and acclimatised to laboratory conditions for at least two weeks prior to the experiment. Fish were kept in five 350 litre aquaria at 17°C and a light regime of 12∶12 h. The cue donor was a pike *Esox lucius* (43 cm total length) from Lake Krankesjön, 20 km east of Lund. The pike was acclimatised in a 150 litre aquarium and fed crucian carp twice a week prior to the experiments.

### Water

The optical properties of the water (tap water) in the experimental arenas were manipulated by adding either bentonite clay, algae (*Scenedesmus* sp.; from a laboratory culture) or brown water, all with pH ranging between 6.6–6.9. The brown humic water was collected from a fish-free pond close to Lund and was filtered (0.1 µm) before being used in the experiment. The effect of clay, algae and brown substances on optical conditions in water is normally measured in different ways, but here we used visual range as a measure of the change in optical condition in the water. Visual range was measured in a glass cylinder (Ø: 6.5 cm H: 43 cm), with a white bottom with a black cross. We added clay, algae or brown water to dechlorinated tap water until the human eye could no longer separate the black cross from the white background in a 40 cm water column. The 40 cm visual range corresponded to turbidity levels of 9 NTU for the clay and 4 NTU for the algal treatment (measured with a LaMotte TC3000 turbidity sensor). The selected visual range and turbidity are comparable to natural condition in lakes exposed to eutrophication or brownification. The chlorophyll *a* concentration in the algal suspension was 346 µg/l, and the absorbance of dissolved organic carbon (DOC) in the brown water was 0.21. Both chlorophyll *a* and brown water were measured in a spectrometer (Beckman DU 800) at 665/750 (chl *a*) and 420 nm (DOC). Effects of treatments on light intensity were measured with a light meter **(**International Light) at a water depth of 5.5 cm (clear water: 11.5; Clay: 9.8, algae: 8.9 and brown: 5.3 µmol m^−2^s^−1^). The light intensity in clear, clay and algae water equals daytime conditions, whereas the lower light intensity in brown water more represents the conditions towards twilight [26]. The spectral properties for the three treatment waters were measured with an Ocean Optics USB2000 spectrometer (HR4000 with a 50 µm slit; measures wavelengths between 200–1100 nm with a precision of 1.8 nm). The slit opening was tilted in a 15 degree angle perpendicular to the water surface, which allows scattered light to enter the slit opening and gives a more representative measure of the wavelength spectrum of light entering the fish eye, compared to if the slit opening was directed towards the light source and the scattering effect was neglected [27]. A halogen lamp (500 W) was used as a light source when we measured the optical properties of the water as well as for lighting the experimental arena during the experiments. In the experiments, a clear water treatment (dechlorinated tapwater, <0.1 NTU) acted as a control for the clay, algae and brown water treatments. To prepare the pike cue water the pike aquarium was cleaned, water exchanged and pike fed with crucian carp for two days. The experiment was performed on day three and four when cue water was taken from the pike aquarium, filtered (500 µm net) and used in the trials.

### Experimental Design

The experiment was performed in a cylindrical arena (diameter 60 cm) with a water depth of 5.5 cm. The shallow water allowed recording of fish behaviours in the turbid/brown treatments using a video camera placed above the arena, whereas fish experienced a reduction in visual range in the horizontal plane. The arena was aerated during the trial. At the start of an experiment we placed a crucian carp in the arena and allowed it to acclimatise for 30 minutes, as a preliminary experiment had shown that carp activity had stabilised by then. After the fish had been acclimatised we started to film its activity and recorded it on video. The activity was recorded for 10 minutes before (pre-stimulus period) and 2 minutes after (post-stimulus period) addition of predator cue water as a pilot experiment showed that the initial response to predator cue started to decrease after 2 minutes. The stimulus of 50 ml predator cue water was added through a plastic tube during two minutes in between the pre- and post-stimulus periods. The water was exchanged and the arena cleaned before each trial. Treatments were replicated 10 times, resulting in a total of 40 carp individuals used, each participating only once in experiments. The study complies with the current laws in Sweden; ethical concerns on care and use of experimental animals were followed under the permission approved for this study (M165-07) from the Malmö/Lund Ethical Committee.

### Video and Statistical Analysis

The video tapes were analysed for swimming activity (total distance moved during a two minutes period) during the pre- and post-stimulus periods, using the behavioural analysis software Ethovision 1.90 (Noldus Information Technology, Wageningen, The Netherlands). A mean of the five two minutes periods from the ten minutes pre-recording were used in the analysis. Relative changes in individual activities between the pre- and post-stimulus periods were calculated as (post - pre)/pre stimulus swimming activity, and these relative changes were tested for differences from no (zero) change with one-sample t-tests for each treatment. Differences in relative change in activity between treatments were analysed with ANOVA and post-hoc Tukey tests. All data adhered to the homogeneity and normality assumptions. An analysis of crucian carp swimming activity during the pre-stimulus period showed no significant difference in base-line activity between treatments (ANOVA, F_3, 36_ = 2.576; p>0.05).

## Results

**Figure 1:**
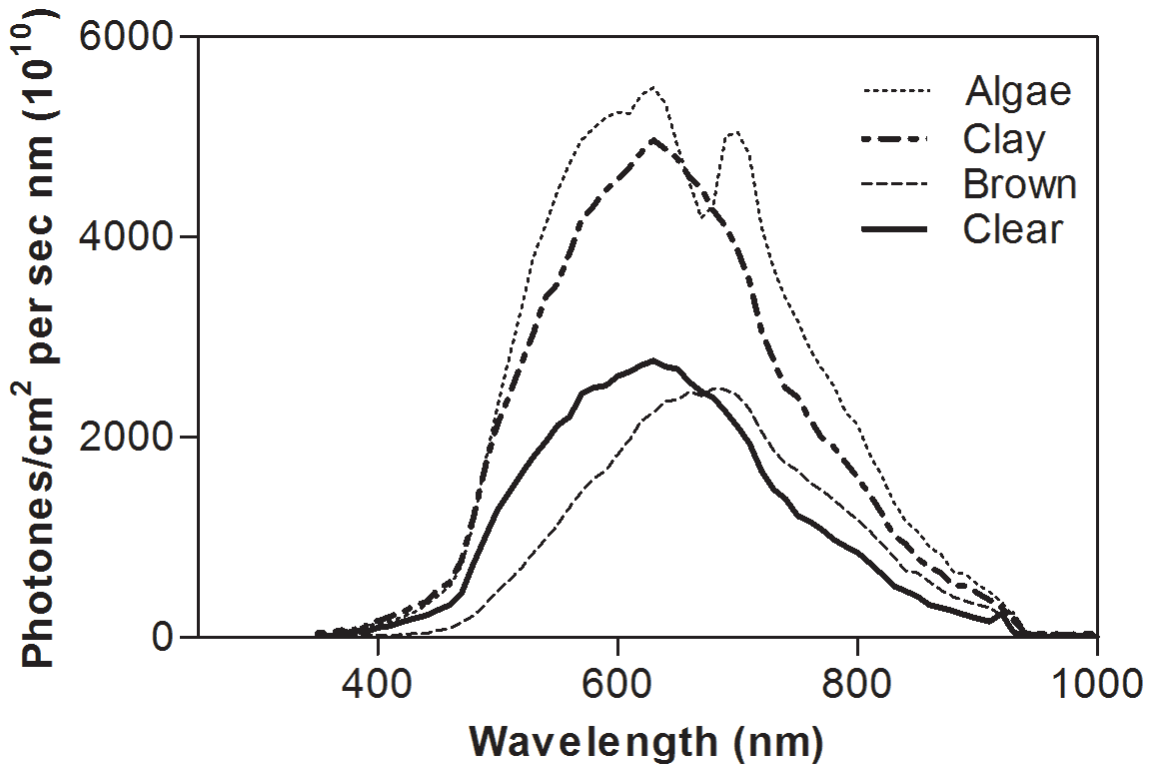
Spectral distribution of downwelling light in clear, algae, clay and humic brown water at 5.5 cm depth.

The clay, algae and brown water treatments reduced light intensity by 15, 23, and 54%, respectively, at a water depth of 5.5 cm. The spectral reflectance analyses showed that the addition of algae and clay resulted in increased scattering, whereas there were no major changes in spectral ranges except an increased absorption of light around 665 nm in the algae treatment, corresponding to the absorbance peak of algal chlorophyll (Fig. 1). In brown water there was reduction of shortwave light in the UV and blue range and an increase of light in the red-infrared area.

**Figure 2:**
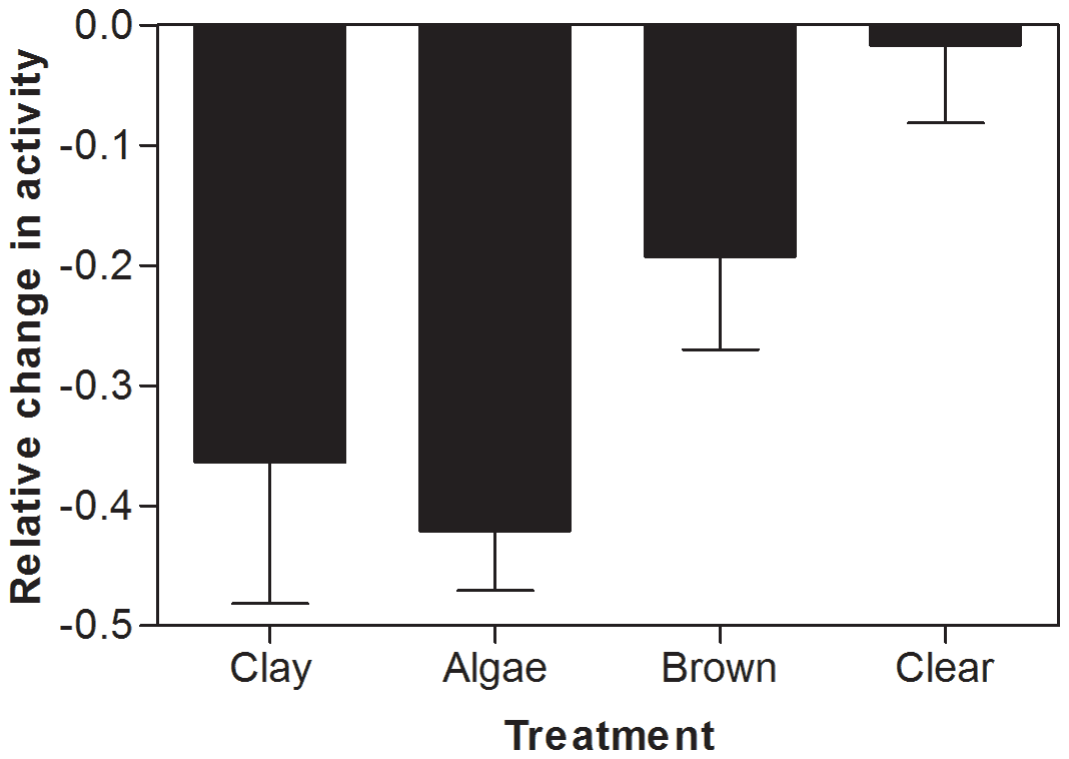
Relative change in swimming activity (mean ± standard error) in crucian carp upon experiencing chemical cues from a pike. Clay, algae and brown water treatments represent a visual range of 40 cm created by the different substances, whereas clear water is a control with no visual deterioration.

Addition of predator cue resulted in a significant reduction in crucian carp swimming activity in clay (t_9_ = −3.090, p = 0.013), algae (t_9_ = −9.395, p<0.001) and brown water (t_9_ = −2.495, p = 0.034), while no change in activity was detected in the clear-water control (t_9_ = −0.261, p = 0.800, Fig. 2). There were also differences between clay, algae, brown and clear treatments in the relative activity change (F_3, 36_ = 4.721; p = 0.007, Fig. 2). The relative change in swimming activity was significantly different between the clear water control and the clay and algae treatments (Tukey HSD, p = 0.021 and p = 0.010, respectively). There were no significant differences between the brown water treatment and the clear water control, or between any of the treatments with deteriorated optical properties (p≥0.300 in all cases).

## Discussion

In this study we found a significant reduction in the activity of crucian carp in response to chemical cues from a pike predator. The response was however context-dependent, with significantly decreased activity in waters with deteriorated visibility, whereas there was no response in activity to predator cues in clear water. A large number of studies have shown that aquatic prey organisms respond to chemical cues associated with predation threat by changing their behaviour (e.g. [12], [28], [29]). In many fish, including crucian carp, specific alarm substances that can be released upon danger, direct injury or indirectly by piscivores being chemically labelled with alarm substances via their diet, elicit predator-avoidance behaviours (e.g. [9], [29], [30], [31]). As the pike in our experiments was fed crucian carp, we believe such alarm substances are involved in the observed activity responses. The cue concentration we used has in earlier experiments failed to elicit threat responses in clear water [9], while in this experiment crucian carp changed their activity in waters with detoriated optical conditions even at these low cue concentrations. Prey fish are able to detect alarm signals at concentrations lower than the threshold concentration needed to elicit a behavioural response [32], [33], and our results indicate that the response threshold changes with visibility conditions. Further, although most studies on the effects of alarm cues have been performed under laboratory conditions a number of recent studies now demonstrate that alarm substances operate also under a wide range of natural conditions [34].

Prey fish may use both visual and chemical cues to detect the presence of predators. It has been argued that chemical and visual cues provide information at different resolution levels, where chemical cues signal the general but spatio-temporally imprecise presence of a predator, whereas visual cues are used at shorter range with high spatial and temporal accuracy [17], [28]. Other studies argue that fish can use chemical cues to make fine-tuned decisions regarding behavioural modifications to predation threat [12]. The large selection pressure for correctly assessing predation risk suggests that using multiple cues to assess local predation risk should be favourable.

The sensory compensation model [17] suggests that vision is the primary source of information about predation threat, and that other cues are used only when the optical conditions in water reduces the reliability of visual information, e.g. poor performance by vision is compensated for by enhanced performance of chemosensory abilities. A basic prediction from the sensory compensation model is that predator chemical cues only initiate a fright response when the information from visual cues is reduced in degraded optical conditions [17]. Our result of no response in crucian carp to predator chemical cues in clear water, but significantly reduced activity in reduced visibility, is in line with the predictions from the sensory compensation model. Sensory compensation has, apart from the minnows studied by Hartman and Abrahams [17], also been demonstrated in diving beetles that did not respond to predator chemical cues in daylight conditions, but showed a strong response to predator cues in reduced light [35]. Diving beetles have well developed eyes and it was suggested that vision is their primary source of information regarding predation threat.

The behavioural response to predator cues differed among treatments with a significant reduction in clay and algae water only. Suspended clay particles and phytoplankton largely affect the optical conditions by scattering incoming light and affecting background contrast, with less of an effect on light intensity. Further, light of specific wavelengths (around 665 nm) is also absorbed by algae, which thus may affect the spectral composition of available light. Studies on the spectral sensitivity of goldfish (*Carassius auratus*), a species that is closely related to crucian carp, have shown a wide spectral sensitivity range (340–720 nm) with three sensitivity peaks; in the UV, shortwave (480 nm) and the longwave (around 650 nm) regions [36]. Changes in spectral composition in the long-wave region in the algae water had no effect on the behavioural response compared to the response in clay water, suggesting that spectral changes are of no importance here. However, the spectral composition was more strongly affected by the addition of brown water with a clear reduction of light in the UV and blue wavelengths. Fish are known to use UV vision in foraging, mate recognition and navigation [37], but no studies have examined the importance of UV vision for detecting predators. Besides changing the spectral composition, the humic substance in brown water also absorbs light, and our measurements showed a 50% decrease in light intensity to 5 µmol m^−2^s^−1^ already at a water depth of 5 cm, i.e. a larger reduction of light intensity than in clay and algae waters. Jachner [38] found that bleak (*Alburnus alburnus*) responded behaviourally to chemical cues from piscivorous pike during daylight conditions only. During night there was no effect of chemical cues, indicating that piscivore-related cues may be perceived as less dangerous during night. The light intensity in the brown water was higher than at night, more resembling light intensities towards twilight conditions. However, both experimental [26] and theoretical [39] studies suggest that a reduction of light intensity to 5–10 µmol m^−2^s^−1^ have significant effect on the reaction distance of a fish, i.e. the brown water to reduce the visual information available for crucian carp compared to the turbid treatments. Thus, the different behavioural response in the brown-water treatment, intermediate and not significantly different from either clear or turbid water treatments, may be due to light intensity. However, humic substances in brown water may also change the chemical properties of predator chemical cues, and thereby affect the ability of prey fish to detect and respond to them. Increased levels of humic acids have been shown to reduce or completely obliterate recognition of conspecific pheromones in goldfish *Carassius auratus* [40] and other fish species [41], [42]. Thus, changes in the chemical environment may also contribute to intermediate behavioural responses to chemical cues in humic, brown-stained water.

In conclusion, the alarm response in crucian carp was affected by the optical conditions in the water. Turbid conditions resulted in a strong behavioural response to chemical cues from pike, compared to clear water conditions where there was no effect. This supports the sensory compensation model [17] that suggests that when one sense is impaired, an organism will compensate by relying on another. The brown water treatment had an intermediate effect on behaviour, which could be either due to changes in light intensity and spectral composition and/or to a disturbance of the chemical senses. The optical condition used in our experiment are already found in many aquatic systems, and with the expected increase from brownification and also eutrophication we can expect even more lakes with short visual ranges. Thus, changing optical conditions in freshwater systems, may affect interactions between predator and prey fish through effects on the chemical communication systems.
